# The Immediate Filling of Pulpless Teeth

**Published:** 1888-02

**Authors:** Louis Ottofy

**Affiliations:** Chicago, Ill.


ARTICLE V.

### THE IMMEDIATE FILLING OF PULPLESS TEETH.

BY LOUIS OTTOFY, D. D. S., CHICAGO, ILL.

[Read before the Odontological Society of Chicago.]

Almost any novel suggestion or practice, which is at variance with established methods or principles, is at first received with diffidence and mistrust; especially if the new practice is not entirely in accord with established laws, rules or principles; hence, when the method of treating pulpless teeth at one sitting, irrespective of previous conditions, was brought to the notice of the profession, naturally the practice was pronounced by many to be irrational and unscientific, certain failure was predicted because it was entirely in opposition to well established physiological laws.

When there is any new method or process suggested, it creates immediately an idea that its application must be universal, and some men are not prone to admit success unless the application proves universal and invariably successful.

This is true not only of immediate root-filling, but of many other practical subjects relating to our profession. Matrices, separators, implantation, bridge-work, and even crown-work, and a number of other practical methods or operations, are, by many, not considered successful because they are not universal in their application. The mind, therefore, should be freed from this impression that the practice about to be advocated in this paper *is* universal. Its sphere, on the contrary, is circumscribed by certain narrow limits, and whenever those limits are passed, the result is, or may be, unfavorable.

There are reasons why the immediate filling of pulpless teeth is justified in this age of steam and electricity, for one of the cardinal principles insuring the favorable consideration of any new invention or novel suggestion is found in its time or labor-saving quality, and it is principally on this ground that immediate root-filling suggests itself to the consideration of the dentist.

As it is customary with all dentists to fill immediately the roots of single-fanged pulpless teeth having an alveolar abscess connecting with the exterior, or those in which the pulp has just been devitalized and extirpated, those classes of teeth are excluded from consideration in this paper. Reference will be made only to teeth which are provided with blind abscesses, or whose periosteum is acutely inflammed, and which it has been customary to treat from two to six times prior to introducing the root-filling. The subject selected for this operation—which has been termed heroic treatment—should be (as in all cases necessitating rapid tissue reproduction, or rapid transformation from pathological to physiological conditions), healthy, robust, and usually young and active ; those provided with nervous and sanguinary constitutions being preferable to ænemic, phlegmatic, and lymphatic constitutions.

Any tooth is a proper one for the operation, but free access to all roots should first be obtained, for thorough cleanliness and dryness are all-important factors. The rubber-dam should be adjusted, the *debris* entirely removed from the cavity before any attempt is made to enter the pulp-chamber, and the root canals, once opened, should never be bored or reamed, nor should any attempt be made to enlarge them; but instead, a good supply of very fine piano-wire instruments should be provided. The first step consists in saturating the entire tooth and cavity with a solution of bichloride of mercury, one part to one thousand of water; then follows the thorough cleaning of the canals with cotton wound on broaches and dipped in chloroform or ether, the object being to dissolve and remove the fats and foreign substances by the aid of these volatile agents. Only very few hairs of cotton should be used, thus preventing a pumping or forcing action toward the end of the root.

These washings should be continued assiduously until neither odor nor color is perceptible. However, in roots where the apex is very large (a fact readily determined by the experienced hand), the cotton receives a slight yellowish tinge, which does not cease, and is no bar in those cases to proceeding with the treatment.

After thorough cleaning, a solution of bichloride of mercury, one part to two hundred and fifty parts of water, is introduced into the root, but not forced beyond the apex. This having been allowed to remain two or three minutes, it is completely removed, and a weaker solution, namely, one part of the bichloride of mercury in one thousand parts of water, is forced into the root and beyond the apex. After a conscientious application of this powerful germicide, the root canal is thoroughly dried, and peroxide of hydrogen is allowed to take its place, which, also, should be pumped with a piston-shaped piece of cotton into every available space within the root and beyond it. If any pus is present, and its presence is indicated by the peroxide of hydrogen, the bichloride of mercury, one in a thousand of water, should again be used; but if no pus is present its use may be dispensed with. The root canals are now carefully dried with hot air, and are then again medicated by winding cotton on a broach, moistening it with eucalyptol, and dipping it into iodoform; this is forced into the roots very thoroughly and conscientiously. While in this condition, the gutta percha dissolved in chloroform is introduced, in the usual manner. Instead of the gutta percha cones, I have been in the habit of making cones of oxyphosphate of zinc; and forcing them into the canals in a semi-hardened condition (either may be used); and acting as a piston, the soft gutta percha should be forced by the cone, thoroughly driving it into every space, irrespective of the fact of its passing through the apex of the root. A filling of gold (if not too large a cavity) may be immediately proceeded with. Any of the plastics may, almost invariable, be introduced at once.

The application of a counter-irritant to the gums is then indicated, which may be either a mixture of equal psrts of tincture of iodine and tincture of aconite root, or an iodine paint, which is iodine dissolved in alcohol, four times the strength of the officinal preparation. In order that either may prove effective, the tissue to which it is applied must be dry. The patient is instructed to return within twenty-four hours in case of trouble. As a general rule, inflammation, sometimes quite severe, of three or four hours' duration, will follow the treatment. When a case, thus treated and filled, is successful, it does not differ in any way from a tooth treated in the usual manner; and, I believe, the liability of recurrence of disease is not more probable than in those subjected to a prolonged course of medication. In a few of my earlier cases the treatment proved unsuccessful, the patient returning the following day with the usual symptoms accompanying the formation of alveolar abscess, but by careful observance of the principles herein laid down, general success follows the practice. While not recommending the method for universal practice, all practitioners will find a number of cases in which it is impossible to continue, or even undertake, the treatment of a diseased tooth, or when, from any reason, the operation must be done immediately, the tooth lost or its treatment entirely abandoned; in these cases, certainly, an attempt to thus save the tooth would be entirely justified.

The following precautions should be observed, *invariably:*

1st. Do not select patients of lymphatic, ænemic, or otherwise sluggish constitutions, but robust, healthy persons. 2d. Use none but absolutely pure and reliable remedies. 3d. Perform each step faithfully, conscientiously and thoroughly before another is taken.

It requires from one-half to one and one-half hours to properly perform such treatment.—*The Dental Review.*

